# Neural Determinants of Sedentary Lifestyle in Older Adults: A Brain Network Analysis

**DOI:** 10.1002/brb3.70085

**Published:** 2025-01-08

**Authors:** Mohsen Bahrami, Jonathan H Burdette, Paul J Laurienti, Barbara J Nicklas, W Jack Rejeski, Jason Fanning

**Affiliations:** ^1^ Laboratory for Complex Brain Networks Wake Forest School of Medicine Winston‐Salem North Carolina USA; ^2^ Department of Radiology Wake Forest School of Medicine Winston‐Salem North Carolina USA; ^3^ Section on Geriatric Medicine, Department of Internal Medicine Wake Forest School of Medicine Winston‐Salem North Carolina USA; ^4^ Department of Health and Exercise Science Wake Forest University Winston‐Salem North Carolina USA

**Keywords:** brain networks, fMRI, number of steps, sedentary behavior, time sitting

## Abstract

**Purpose:**

The prevalence of sedentary lifestyles (SL), which includes both high volumes of extended sitting behavior and a low volume of steps accumulated across the day, among older adults continues to rise contributing to increases in associated comorbidities and the loss of independence. The social, personal, and economic burdens are enormous. In recognition of the health implications of SL, current public health physical activity guidelines now emphasize the complimentary goals of sitting less by moving more. We recently completed a 6‐month weight loss (WL) study followed by 12 months of reduced contact to examine weight regain in older adults with obesity. One of the treatment conditions involved WL + a day‐long movement intervention that explicitly targeted reducing sitting time and increasing steps across the day (SitLess).

**Method:**

The goal of the current study, using baseline fMRI and accelerometry data from 36 participants and advanced machine learning tools, was to determine if we could identify complex brain circuits underlying variability associated with changes in sitting time and daily steps during the 6‐month intensive phase among participants randomized to the WL + SitLess treatment condition. Models generated from these analyses produced accuracy in predicting pre–post change in both measures that exceeded 92%, suggesting a critical role for the identified brain subnetworks in explaining variability in these outcomes in response to the intervention. The identified networks comprised regions, predominantly in the default mode and sensorimotor networks, that have been extensively linked to self‐regulation and decision‐making.

**Finding:**

These results provide insights into the theoretical basis of SL for older adults and in the design of future intervention research.

## Graphical abstract

In this study, we used brain imaging data (fMRI) to identify brain networks that mediate sedentary behavior during an intervention. We found that networks comprising key regions in well‐established networks, including the default mode network, play key roles in mediating the sedentary behavior. These networks allow for predicting the success or failure in improving the sedentary behavior with over 90% accuracy.

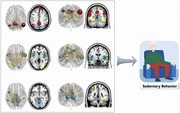

## 1 Introduction

Sedentary lifestyles (SL), which involve the combination of extended sitting and a low volume of steps across the day, independent of time spent in moderate‐to‐vigorous intensity physical activity (MVPA), have increasingly been linked to multiple important adverse health outcomes (Thorp et al. 2011; Tremblay et al. 2017). Recent reports indicate that 27.5% of the global adult population (Guthold et al. 2018) and over a third of the US population (Du et al. 2019; Guthold et al. 2018) lead a sedentary lifestyle. Furthermore, the prevalence of sitting behavior among older adults is strikingly high at > 65% (Harvey, Chastin, and Skelton 2013). This places enormous economic burden on private and public healthcare systems with an estimated cost of $53.8 billion per year (Ding, Lawson, and Kolbe‐Alexander 2016). Moreover, societal indicators of energy expenditure show a remarkable increase in SL over the past several decades (Owen et al. 2010), raising even further concern about associated health and economic consequences.

Structured exercise (Dempsey, Biddle, and Buman 2020), particularly walking interventions (Gomes‐Osman et al. 2018), has been extensively studied and recommended to combat the negative impact of insufficient physical activity (PA) in older adults. Although such interventions have proven efficacy in the short term (Pahor et al. 2014; Snowsill et al. 2022), their effects typically dissipate following the end of formal treatment (McEwan, Rhodes, and Beauchamp 2022). This may be due to a noticeable lack of understanding about the determinants of an SL (Morris et al. 2022; Owen et al. 2011; Spence, Rhodes, and Carson 2017) as a distinct construct from MVPA (Spence, Rhodes, and Carson 2017). Counteracting SL is not solved by simply increasing MVPA via exercise (Spence, Rhodes, and Carson 2017; van der Ploeg and Hillsdon 2017). Rather, the demands of modifying SL are highly unique from those required to engage in structured exercise (EX). Where exercise requires the individual to use substantial motivation once per day on most days per week, altering SL requires individuals to self‐monitor sitting behaviors continuously throughout the day and to develop an awareness of their behavioral patterns, to frequently act to break up SB, and to sustain this motivation continuously, even when fatigued or experiencing poor affective states (Maher and Dunton 2020). It is also important to note that, in response to adopting EX, many older adults reduce time spent in other forms of PA and sit more often and for longer periods of time, thus displaying little improvement in total energy expenditure (Thompson et al. 2014). As such, research on understanding the mechanistic determinants and mediators of SL as a distinct construct from EX is needed for a meaningful shift in designing efficient interventions.

The critical role of cognitive processes in regulating SL and maintaining exercise interventions has been well established in the literature (Cheval et al. 2020; Conroy et al. 2013; Szczuka et al. 2021). For instance, self‐efficacy, defined as the individual's belief in their capacity to engage in actions required to achieve a specific goal (Bandura, Freeman, and Lightsey 1999), has been associated with decreased sitting behavior (Szczuka et al. 2021), with some studies having shown reduced sitting by targeting self‐efficacy mechanisms (Falk, O'Donnell, and Cascio 2015). However, to our knowledge, no research has explored neural phenotypes as mechanistic determinants underlying these cognitive processes, which presumably mediate changes in SL. Understanding such mechanistic determinants is a crucial step toward designing interventions for sustained behavioral change (Falk, O'Donnell, and Cascio 2015; Morris et al. 2022). Specifically, knowledge about specific neural circuits that mediate daily SL can be subsequently translated into theories on brain–behavior adaptations (Brand and Cheval 2019; Conroy et al. 2013) to provide a rigorous foundation for developing targeted treatments and individual‐based interventions stemmed from these neural determinants.

To fill this important gap, a new line of research has evolved using neuroimaging data to explore the neural underpinnings of SL. In a recent study, Morris et al. (2022) found that functional connectivity between the anterior cingulate cortex and supplementary motor areas, as well as the connectivity between anterior insula and the left temporoparietal/temporooccipital junction, is strongly associated with interindividual differences in response to a walking intervention. The insula and particularly the anterior cingulate cortex are well‐known areas for reward‐based decision‐making processes (Guo et al. 2013), and thus, the altered functional connectivity reported by Morris et al. (2022) may explain the difference in inhibitory control toward regulating effort in response to the intervention. The role of such regions in regulating SL has been reported in other studies as well (Falk, O'Donnell, and Cascio 2015; Jackson, Gao, and Chen 2014). In a very recent study, Falck et al. (2024) used task‐based fMRI data from 26 participants participating in a 6‐month intervention to examine neural determinants of sitting behavior and PA. By examining the activity of brain regions, they showed that PA is regulated by the activity of several important regions, such as the anterior cingulate, frontal pole, and precuneus, across overlapping networks, including the default mode, frontoparietal, and cingulo‐opercular networks. Although this result for PA suggests a complex neural activity during executive control, such neural determinants were not found for sitting behavior, underscoring further research on neural determinants of SL. Mechanistic determinants of SL, and particularly those that explain the change resulting from interventions, have yet to be identified. Specifically, current neuroimaging studies have focused on examining how neural activity or static patterns of individual connections are associated with sitting behavior or PA in cross‐sectional analyses (Pindus et al. 2020; Voss et al. 2010, 2016). Given that the brain is a dynamic multiscale system (Lurie et al. 2020), we hypothesized that dynamic patterns of complex circuits in the brain can provide new insights into longitudinal changes in sitting time (a marker of SL) and number of steps (a marker of PA volume) that occur as a result of a 6‐month active intervention, one specifically designed to target these metrics. Using dynamic networks constructed from fMRI data and a tensor‐based analytical framework, we first extracted complex circuits that explain the variability among different individuals and then used those circuits to predict changes in sitting time (st) and number of daily steps (ns). We have previously utilized this promising approach to predict weight loss (WL) (Mokhtari et al. 2018), in which we approached an accuracy exceeding 95% with identified networks showing significant association with WL even across independent samples (Burdette et al. 2022, 2020). Moreover, the main goal of this study was to identify dynamic networks that predict pre–post change in SL using a data‐driven approach to allow for finding potentially new regions as well. The utilized approach in this study provided a unique framework to achieve this due to the following reasons: (i) It is a data‐driven approach that allows identifying novel networks; (ii) the networks are extracted from dynamic patterns of brain connectivity; and (iii) it allows for predicting the pre–post change in SL from brain scans at baseline that could be subsequently used for determining whether individuals would succeed or fail at modifying SL prior to starting the intervention.

## 2 Materials and Methods

### 2.1 Participants

A sample of 71 older adults with obesity underwent MRI scanning as part of a weight‐regain study named Empowered with Movement to Prevent Obesity and Weight Regain (EMPOWER; ClinicalTrials.gov identifier: NCT02923674). All participants recruited for EMPOWER met the following requirements: 65–85 years of age; a body mass index (BMI) between 35 and 45 kg/m^2^; low active, defined as < 20 min/day of self‐reported exercise (Fanning et al. 2018); and no evidence of cognitive impairment (Nasreddine et al. 2005). More details about the methods can be found in Fanning et al. (2018). Participants who were recruited for scanning had no contradictions for the MRI scan and completed an in‐person screening visit and a 45‐min MRI scan after an overnight fast. MRI data were collected at baseline prior to initiating the interventional treatment. Of the 71 participants who completed the MRI scans, 4 participants were removed because of excessive head motion, and 31 further participants were removed due to lack/insufficient data for SB measurements at either baseline or the 6‐month follow‐up (this is described in detail in the next section). Therefore, the final sample used in the study included 36 participants. This sample represented the larger cohort who underwent MRI scanning in key demographics, including age, baseline weight, and male/female ratio (Table S1).

### 2.2 SitLess Intervention

Participants from the parent study were randomly assigned to three different interventions, including WL + EX; WL + a novel daily movement intervention (SitLess); and WL + EX + SitLess. In this study, we used those who were randomized to the WL + SitLess intervention (Nicklas et al. 2014) as a primary goal of the parent study was to determine whether adding the SitLess intervention to the frequently used WL interventions improved the outcomes of such standard interventions. Intervention procedures have been described in detail (Fanning et al. 2018, 2022). Briefly, all participants received a Fitbit activity monitor at least 2 weeks before starting the interventions, and these data were streamed into a study‐specific mobile health (mHealth) app to provide real‐time feedback on patterns of activity specific to each arm. More details about this app could be found in Fanning et al. (2018, 2022). The SitLess intervention was aimed at improving the number of daily breaks in sitting (at least a 25% improvement) as well as the number of daily steps (an increase of at least 3000). Participants were provided with instructions to reduce sitting time through frequent bouts of activities of various intensities (e.g., stand and engage in light movement while watching television) and within the community (e.g., identify opportunities for active transport). Participants attended weekly group‐mediated sessions led by a behavioral specialist and registered dietician, and these sessions covered topics related to the importance of reducing sitting and increasing activity for health. The sessions also provided training on skills required for successful behavior change (e.g., goal setting and revision, the influence of thought patterns on behavioral choices) and allowed groups to review their weekly progress, troubleshoot challenges, and set new goals. The mHealth app provided visual feedback on patterns of activity and inactivity via a timeline bar wherein periods without steps were displayed in blue, and periods with steps were displayed in green. Participants also received three daily “periodic” step goals, which encouraged participants to make progress toward their daily step goal in each of three periods: before lunch, between lunch and dinner, and after dinner.

### 2.3 MRI Scanning

Participants were scanned in the morning following an 8‐h overnight fast. Each scanning session included anatomical imaging, resting‐state fMRI, and a food‐cue visualization fMRI scan. This study is focused on the resting condition. During this condition, participants were instructed to keep their eyes open and focus on an on‐screen fixation cross for 7.2 min. MRI data were collected using a Siemens 3T Skyra MRI scanner equipped with a 32‐channel head coil. Participants were positioned to visualize a rear projection screen and received MRI earplugs to protect their hearing during scanning. T1‐weighted structural images were first acquired in the sagittal plane using a single shot 3D MPRAGE GRAPPA2 sequence with the following parameters: TR = 2.3 s, TE = 2.95 ms, flip angle = 9°, resolution = 1.1 × 1.1 × 1.2 mm^3^, and number of slices = 176. Functional scans were acquired using blood‐oxygen‐level‐dependent (BOLD) T2^*^‐weighted images and single gradient echo‐planar imaging (EPI) sequence with the following parameters: TR = 2.0 s, TE = 25 ms, flip angle = 75°, acquisition time = 7.23 min, resolution = 4 × 4 × 4 mm^3^ (adjusted to 4 × 4 × 5 mm^3^ after scanning the first 23 participants as the images with the original voxel size failed in covering the entire brain in several participants), and number of volume = 217.

### 2.4 Image Processing

#### 2.4.1 Preprocessing

We first completed a series of image preprocessing analyses using Statistical Parametric Mapping 12 (SPM 12, Welcome Trust Center, London, UK: www.fil.ion.ucl.ac.uk/spm/), advanced normalization tools (ANTs) (Avants et al. 2011), and in‐house MATLAB scripts. The preprocessing included: (i) segmenting the T1‐weighted structural images to remove skull tissue (skull stripping) and creating a brain tissue mask, and segmenting the remaining image into gray matter, white matter, and cerebrospinal fluid (Ashburner and Friston 2005); (ii) manual cleaning of the generated brain mask in MRICron software (https://www.nitrc.org/projects/mricron) to correct any misclassified voxels; (iii) warping the skull‐stripped T1‐weighted structural images to the Colin brain template (Schmahmann et al. 1999) through ANTs normalization; (iv) transforming the Shen functional atlas (Shen et al. 2013) to each participant's native anatomical space using the inverse of the resulting transforms. The atlas was resliced and co‐registered to match the functional data; (v) removing the first 20 volumes. This resulted in voxel time series with a length of 197; (vi) correcting for slice‐time differences; (vii) removing the regional spurious signals caused by participant head motion through running ICA‐AROMA (Pruim et al. 2015); (viii) removing the low‐frequency drift and physiological noise with a band‐pass filter (0.009–0.08 HZ); and (ix) regressing out the motion parameters with 6 dof and mean signals of the three tissue types, including gray matter, white matter, and cerebrospinal fluid (Biswal et al. 1995). As we warped the atlas into participant's anatomical space, all subsequent analyses were performed in this space. After completing the preprocessing steps with a rigorous quality control for misclassification of tissue segments in the corresponding sections, we extracted the mean time series of each region in the Shen atlas. Time series served as our final preprocessed ROI time series (signals) for all subsequent analyses.

#### 2.4.2 Functional Network Generation

We used a sliding window correlation (SWC) approach with a time window of fixed length W to generate a series of dynamic brain networks for each participant. We used a modulated rectangular window due to its superior performance (Mokhtari et al. 2019). The time points within the window were used to compute the pairwise Pearson correlation for all pairs of 268 ROIs, and then by moving the window across the time and repeating this procedure, 3D data representing a series of correlation matrices across time were obtained. These data were represented by a third‐order tensor, **ℭ** $\in R^{N\times N\times T}$, where the first two dimensions were connectivity with N=268 (number of ROIs), and the third dimension was time with $T=T^{\prime }-W+1$ ($T^{\prime }=197$—length of the preprocessed ROI time series, W= sliding window size). We used a fixed minimum length of 61TRs (W=61) for all subsequent analyses to further ensure capturing the lower frequency components and due to the established performance of this window length in our previous study (Mokhtari et al. 2018). This window length also avoids spurious fluctuations that can occur when the *W* is less than $1/f_{min}$ (Leonardi and van de Ville 2015), where $f_{min}=0.008$. We used a shift size of 1TR for highest temporal resolution (Mokhtari et al. 2018; Shakil, Lee, and Keilholz 2016). Using a proportional thresholding approach (Garrison et al. 2015), the correlation matrices at each window were thresholded to maintain the top 10% of the strongest connections for that window (Mokhtari et al. 2018).

### 2.5 Connectivity Tensor Decomposition

As pointed out in Section 1, we hypothesized that dynamic patterns of complex circuits in the brain may serve as important mechanistic determinants for a successful behavioral change—that is, improvement in the daily sitting time (st) and number of steps (ns) following the 6‐month SitLess intervention in this study. Previously, we have demonstrated the promise of tensor decomposition methodologies in extracting such dynamic patterns with consistent performance across other modeling parameters such as window length (e.g., 71TR and 81TR) and density threshold (20%, 30%, etc.) (Mokhtari et al. 2018). In this study, we used the same tensor decomposition methodology to reduce the complex connectivity tensors into meaningful network components prior to using them in our prediction analysis. This allowed for a substantial dimensionality reduction of the higher dimensional data into lower dimensional data by removing redundant data and providing the network components that explain the variability across the older adults. The tensor‐based methodology used in this study is a data‐driven approach, suited for analyzing dynamic brain networks, that allows identifying components/networks that explain the highest variability across brain connectivity, time, and participants. As in our previous study, we used a higher order singular value decomposition (HOSVD) approach to decompose our connectivity tensors and reduce the dimensionality of our data (de Lathauwer, De Moor, and Vandewalle 2000). Below, we have provided more details.

We performed two separate sets of analyses for the change in st and ns. Here we briefly explain our modeling procedure for the change in st, but we used the exact same procedure for change in ns. For each participant, i, we first obtained the longitudinal change in st ($st_{i}$) between baseline and 6‐month follow‐up using the following equation:

$$st_{i,change}=st_{i,6-months}-st_{i,baseline}$$

where $st_{i,6-months}$ and $st_{i,baseline}$ represent the sitting time after the 6‐month intervention and at the baseline, respectively, for participant *i*, and $st_{i,change}$ represents the change in sitting time for participant *i*. Note that *i* = 1,2,3,…,36, as we had 36 participants. After obtaining $st_{i,change}$ for all 36 participants, we determined the median and assigned each participant to either low‐ or high‐change group, with respect to the median value. Table 1 presents the average values (±std) across the participants on the basis of this classification. Note that participants who were assigned to the low‐st group could fall into the high‐ns, and vice versa. We, thus, performed two different sets of analyses for st and ns. We then randomly split our participants into training (78%−28/36) and testing (22%−8/36) subsets with equal number of participants from each group (i.e., low/high). The individual connectivity tensors across the $M_{Trn}$ ($M_{Trn}=28$) training samples were concatenated to construct a fourth‐order connectivity tensor, $C_{Trn}\in R^{N\times N\times T\times M_{Trn}}$. Using tensor algebra and the same methodology as in Mokhtari et al. (2018), we obtained the reduced‐rank connectivity tensors of the training and testing samples, $C_{rTrn}\in R^{R\times R\times T\times M_{Trn}}$ and $C_{rTst}\in R^{R\times R\times T\times M_{Tst}}$, where R represents the minimum number of components that captured over 85% of variance. Our results showed that we can capture over 85% of variance with R=17 (see Figure S1). We averaged each tensor across time to obtain the summarized reduced‐rank R×R connectivity tensor for each participant. Such reduced‐rank connectivity tensors with embedded time information explain the variability across participants. We, thus, used the final R×R connectivity tensors for our prediction analysis. Note that each component can be represented in the brain space as detailed in Mokhtari et al. (2018). For more technical details and equations, please see the Supporting Information section and our previous study (Mokhtari et al. 2018).

**TABLE 1:** Average daily sitting time (st) and number of steps (ns).

Sedentary measure		Low change (N=18)	High change (N=18)
Sitting time (min)			
	Baseline ($st_{m,baseline}$)	535.48±90.09	603.81±140.45
	6 months ($st_{m,6-months}$)	598.61±117.21	534.98±133.23
	Change ($st_{m,\;change}$)	63.14±68.91	−68.83±36.33
Number of steps			
	Baseline ($ns_{m,baseline}$)	7172.6±3040.8	6243.4±2116.1
	6 months ($ns_{m,6-months}$)	6463.4±2390.6	9197.2±2283.0
	Change ($ns_{m,change}$)	−709.1±1823.6	2953.7±1119.8

*Note*: Subscriptmdenotestheaverageacrossparticipantsinthistable.

### 2.6 Prediction Analysis

We used the reduced‐rank connectivity tensors to predict the change in st and ns (both as binary variables—low/high) in two separate sets of analyses. We used R×R connectivity tensors of training samples to train a linear SVM (Cortes and Vapnik 1995), as implemented in LIBSVM toolbox (Chang and Lin 2011), and tested the performance of the trained model on the R×R connectivity tensors of testing samples. To avoid potential bias or overfit, we did 100 random subsampling cross validation, in which all participants were randomly assigned to the training (28/36) and testing (8/36) subsets with equal group sizes in both the training and testing subsets (i.e., we did 100 analyses with random assignments of participants into training and testing subsets). We also saved the 100 random permutations and used the same permutations for all subsequent analyses of st and ns to better compare the results. The performance of our model was assessed by averaging the results across the 100 permutations. During each permutation and for each analysis, a support vector machine (SVM) model used the reduced‐rank R×R connectivity tensors of the training samples (R=17) as a 289×1 feature vector to find a maximum‐margin hyperplane between the two groups by optimizing a weighted (/parametrized) combination of the 289 features. The learned weights were then used to predict the labels associated with the reduced‐rank R×R connectivity tensors of the test samples. The learned weights for the features can be represented by a weight matrix, $W\in R^{17\times 17}$. We also did additional analyses with random group assignments (i.e., not based on the pre–post change in st/ns) to further ensure that our prediction performances were not random or biased. For the random group analyses, we did the same analyses with the same random permutations but with the labels of the entire data (including training and testing samples) permuted between the two groups prior to the prediction analyses. For each permutation, we determined the accuracy, sensitivity, and specificity of the test samples of that permutation. The final values for each performance measure were obtained by averaging across the 100 permutations. For more technical detail, see the Supporting Information section. We specifically used a linear SVM due to our previous success in using this approach, but, more importantly, to put the highest discriminatory power on the extracted features and to avoid overfitting due to our small sample size. A simple model with excellent prediction performance indicates that input features (mapped as brain networks on the brain) will have the highest contribution toward that prediction. We did not use validation data for hyperparameter tuning as the model that we used (linear SVM) did not require hyperparameter tuning due to its simplicity while still providing excellent performance (due to the high discriminatory power of the input features).

## 3 Results

### 3.1 Tensor Decomposition Components

**FIGURE 1:**
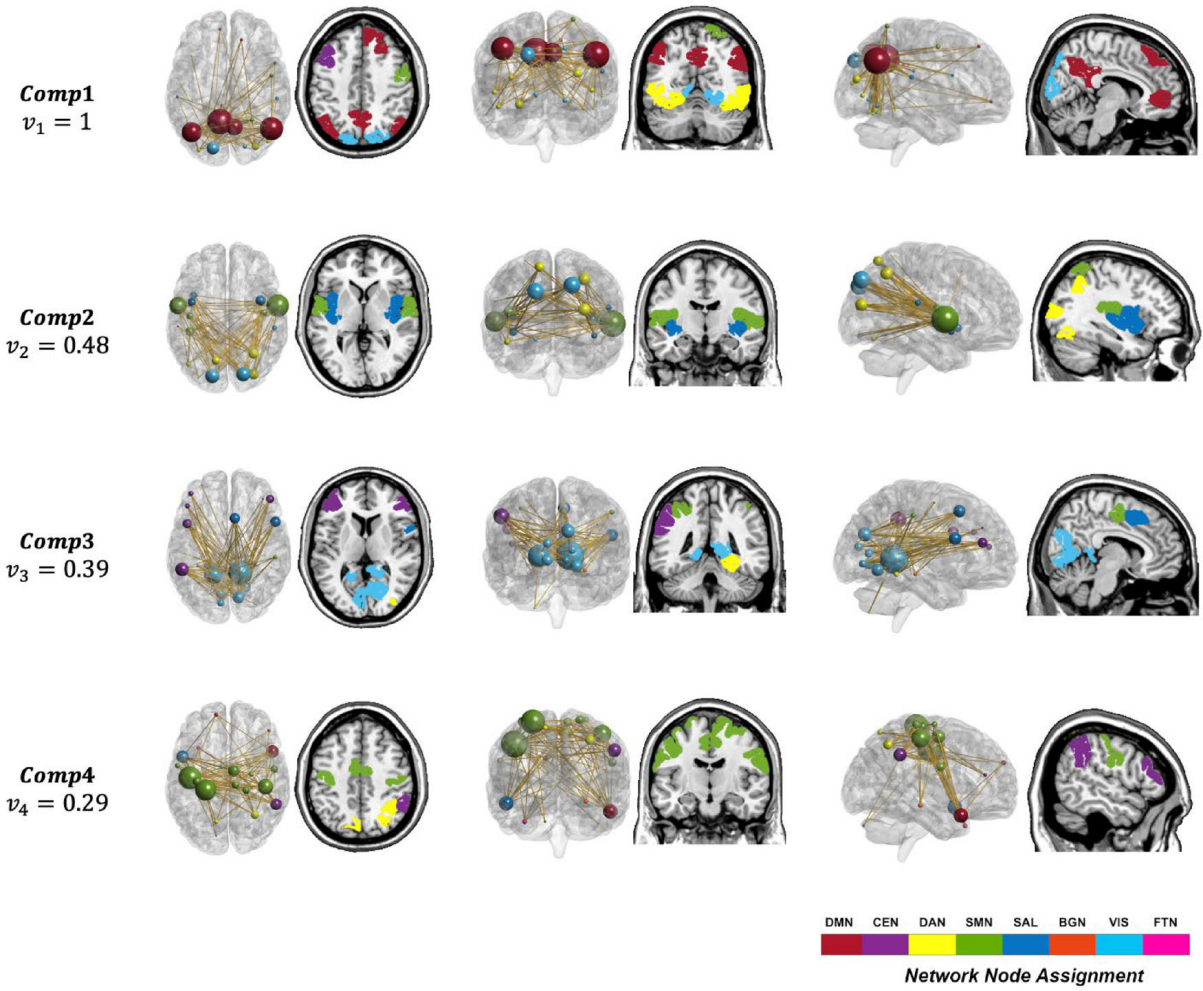
Top‐ranked components in the brain space. The network components constructed from the first four eigenvectors carrying the highest variance are shown in this figure. The singular vectors were first averaged across the 100 permutations and then mapped to the brain space. Networks in this figure show the top 1% of the strongest connections for each singular vector. The axial, coronal, and sagittal brain slices depicting anatomical node locations are shown along with network maps viewed from the same perspective. The size of each node in the network maps represents the number of connections of that node and the color represents its belonging to one of eight networks covering the entire brain: Default mode network (DMN), central network (CEN), dorsal attention network (DAN), sensory motor network (SMN), salience network (SAL), basal ganglia network (BGN), visual network (VIS), and frontotemporal network (FTN). The normalized variance for each component is shown on the left. The normalized variance dropped below 0.25 from Component 5.

Figure 1 shows the Top 4 network components that explain the highest variability in dynamic networks among older adults (the normalized variance from Component 5 dropped below 25%). The networks shown in this figure were reconstructed by their corresponding singular vectors and averaged across the 100 permutations (Mokhtari et al. 2018). We have also shown the normalized variance explained by each component. Networks shown in this figure were thresholded to maintain the top 1% of the strongest connections.

As detailed in Mokhtari et al. (2018), each network component shown in Figure 1 should not be considered a set of independent connections and rather should be considered a complex circuit in which connections show complex interactions across time and participants. In other words, each component comprises a set of interactions that captures the variability across time and participants and is thus a critical set of interactions embedded in the complex high‐dimensional data. To put the highest discriminatory power on interindividual differences, we used the averaged components across time. As such components, with embedded dynamic information, are used for our prediction analysis, a high performance in predicting the desired outcome demonstrates the importance of the components in explaining the interindividual variability in the desired outcome, that is, the pre–post change in st and ns in this study. Interestingly, our components included regions with an established importance for regulating sedentary behavior habits in the literature. Below, we have provided a list of important anatomical brain regions from each component.

***Comp1***. Key regions in the default mode network (DMN), including angular gyrus, posterior cingulate cortex, medial cingulate cortex, precuneus, and medial occipital regions.
***Comp2***. Key regions in the sensory motor network (SMN), including temporal superior cortex, Rolandic, and occipital regions, as well as key regions in the dorsal attention network (DAN) and salience network (Sal), such as insula.
***Comp3***. Key regions in the visual cortex network (VIS), including linguistic, fusiform, and cuneus areas, as well as few regions in the DAN and central executive network (CEN).
***Comp4***. Key regions in the SMN, including superior motor areas, precentral and postcentral areas, and medial cingulate cortex and portions of the DAN and CEN.

The listed regions above had the highest number of interactions with other brain regions in the 99th percentile of the connection strength distribution, which demonstrated their substantial contributions toward a successful prediction of variability in the longitudinal change of st and ns. This will underscore the critical role of such regions in regulating the sedentary behavior habits. We will elaborate on the intuition and implications of our network components and regions with respect to the literature in Section 4.

### 3.2 Prediction Performance

**FIGURE 2:**
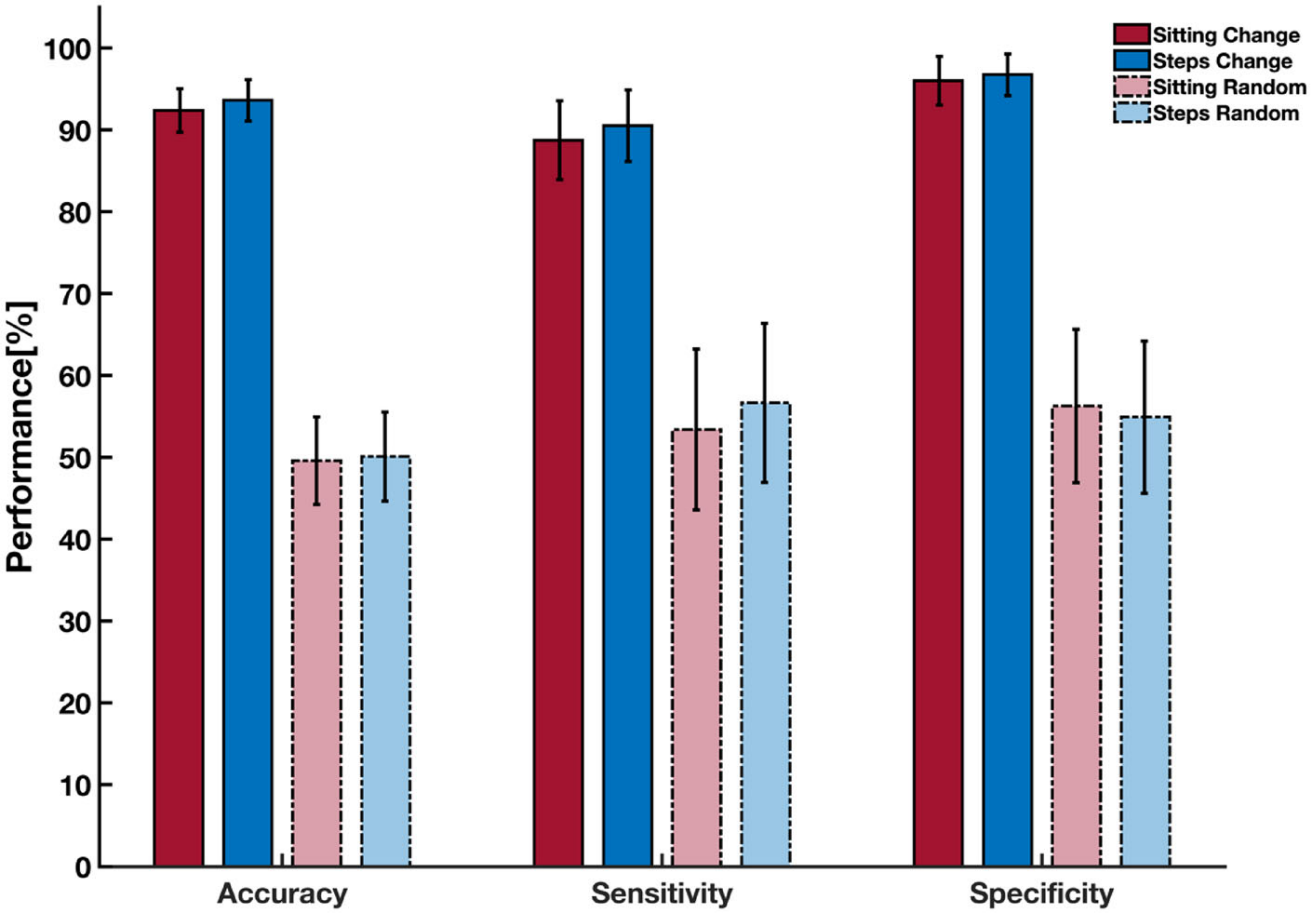
Average prediction performance across 100 permutations. Average accuracy, sensitivity, and specificity, as well as the average cross‐entropy are shown for the main analyses and the analyses with random group assignments. The average and the standard deviation across the 100 permutations are shown in this figure. As illustrated by this figure, the linear SVM has an excellent performance in predicting the pre–post change in both sedentary behavior measures, with superior performance in predicting the samples from the high‐change group for both measures.

The performance for predicting change in st and ns via reduced‐rank connectivity tensors is shown in Figure 2. This figure shows the performance for random assignment of participants into low and high groups as well. Our results indicate an excellent performance with an average accuracy of 92.37±8.86(mean±std), and 93.62±8.42, in predicting the pre–post change in st and ns among older adults, respectively. Our sensitivity (st: 88.75±16.04, ns: 90.50±14.56) and specificity (st: 96.00±9.87, ns: 96.75±8.45) results indicate a better performance in predicting the change in participants who exhibited a higher longitudinal change after the 6‐month intervention. Note that the performance for the analyses with random group assignments did not exceed chance for change in either st (49.59%) or ns (50.09%).

**FIGURE 3:**
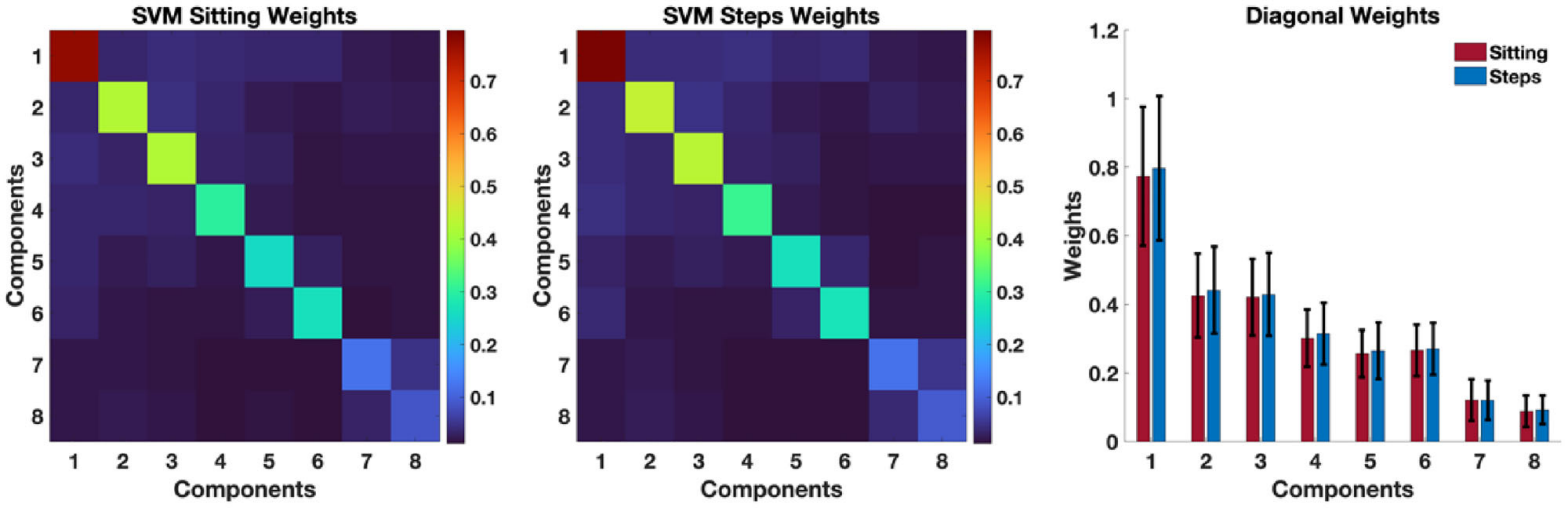
SVM weights for predicting pre–post change in st **and** ns. Average SVM weights across the 100 permutations for predicting pre–post change in st and ns for the first 8 components. The bar plot shows the weight magnitude of diagonal elements. As this figure shows, the SVM weights for predicting pre–post change in ns are slightly greater than those of st, which may explain the slightly higher accuracy in predicting ns. However, this difference did not reach statistical significance.

As discussed earlier, SVM assigns a weight to either one of the 289 features of the reduced‐rank connectivity tensors during the training phase, which can be shown as a 17×17 weight matrix. The diagonal elements of this weight matrix are associated with the individual eigenvalues, and off‐diagonal elements are associated with the interactions among eigenvalues. We have shown the weight matrix, averaged across the 100 permutations for the first 8 components, in Figure 3 to better show the contribution of each component toward prediction results. As this figure shows and as expected, the individual components have much stronger weights and thus play a much stronger role in separating the two groups. The weight matrix also demonstrates the substantially higher contribution of the first individual components, which includes key regions from the DMN, toward separating the two groups. All individual components had larger contributions (greater weights) in predicting the change in st than ns. Moreover, Figure 3 shows *slightly* greater weights of the individual components in predicting ns than st which may explain the *slightly* higher accuracy in predicting the ns than st (93.62 vs. 92.37). However, these differences for both weight contributions and prediction performances did not reach statistical significance.

## 4 Discussion

There is little doubt that extended sitting behavior and a low volume of steps accumulated throughout the day (SL) are important to human health, with SL deserving attention as a distinct class of behavior from EX (Vallance et al. 2011). Historically, theories and conceptual models of sitting behavior and PA have involved: (a) decision‐based frameworks such as Social Cognitive Theory that encompass conscious, intraindividual processes (Bandura 1997; Motl et al. 2019); (b) ecological models that include multiple levels of influence, including intraindividual, social, organizational, environmental, and public policy (Sallis, Owen, and Fisher 2015); and (c) dual process models, which underscore the joint contribution of conscious processes and the nonconscious influence of habits (Maher and Conroy 2016). Note that an implicit assumption of the ecological approach is that nonconscious processes such as conditioned responses to environmental forces play a key role in determining both sitting behavior and the accumulation of daily steps. Despite recognition that both conscious and nonconscious processes likely co‐determine these behaviors, a major limitation of existing conceptual frameworks is that they do not provide insight into the neural architecture underlying these processes. The current study on older adults was designed to evaluate whether neural networks derived from resting state scans collected prior to the onset of a behavioral intervention designed to reduce sustained SL by promoting stepping throughout the day could predict the magnitude of observed changes in these outcomes. As we hypothesized, we were able to derive circuits that explain considerable variability in sitting behavior and daily stepping for different individuals and then use these circuits to predict change over a 6‐month interval of time. It is important to note that network components used as input data to our machine learning approach explained the highest variability across participants (averaged across time) and thus were associated with the variability associated with changes in both outcomes.

Our first network component capturing the highest variability across participants was dominated by interactions among key regions from the DMN, including angular gyrus, medial and posterior cingulate cortex, precuneus, as well as medial occipital regions. Although studies indicate that the DMN can play a role in nonconscious input to self‐efficacy (Wang et al. 2022), which is known to mediate sitting behavior and PA, the DMN has a prominent role in interactions with other networks involved in higher level cognitive tasks (Smallwood et al. 2021) and the self‐regulation of these behaviors (Andrews‐Hanna 2012; Buckley et al. 2014). Self‐regulatory capacity is a well‐known determinant of SL (Buckley et al. 2014). As noted in Buckley et al. (2014), “sedentary behavior may be partially a reflection of self‐regulatory failure resulting from an increased sensitivity to the rewarding effects associated with sitting and inactivity‐related cues,” which fall in the category of conditioned responses. Therefore, this first network component appears to reflect the critical role of nonconscious, self‐regulatory mechanisms arising from the DMN. This finding is in line with meta‐analyses indicating that sitting behavior is largely controlled by nonconscious processes (Compernolle et al. 2019). The third network component, which was dominated by interactions among visual network areas and regions in the DAN, captures brain connectivity that is also linked to nonconscious processes known to affect self‐regulation (Rueda, Posner, and Rothbart 2004).

Brain regions captured by the second, and particularly the fourth network component, were dominated by interactions among areas of the brain known to be central to motor control and sensory processing. These include the superior motor areas, precentral and postcentral gyri, medial cingulate cortex, and, to some extent, the dorsal attention and salience networks. Temporal interactions among sensorimotor areas and the insula, a major player in the salience network, have been linked to decision‐making (Harris and Lim 2016). An increasing number of studies support the position that an inherent feature of decision‐making processes is that they are driven by a principal designed to conserve physical effort (Cheval et al. 2018; Harris and Lim 2016; Klein‐Flügge et al. 2016). Again, these processes are nonconscious. The dynamic interactions of sensorimotor areas with mainly the cognitive systems in the brain, which guide decision making, may well represent a network bias for SL; specifically, resistance to the message to “move more and more often throughout the day!”

The results of this study advance knowledge on the theoretical underpinning of sitting behavior and daily steps that has important implications for future intervention research. First, these data reinforce the important role that habit and thus nonconscious processes play in determining older adults’ proclivity for SL and in their resistance to behavior change (Verplanken and Orbell 2022). The component analyses on network structures provide novel insight into the association between connectivity within the DMN and failure to adhere to intervention goals that targeted decreasing sitting behavior and increasing stepping behavior. As we have described above, this phenotype likely identifies individuals with deficits in self‐regulation; it would be interesting to investigate whether these deficits generalize to other behaviors. In addition, the component analyses revealed that self‐regulatory failure was associated with connectivity within sensory and motor structures. Whether *stronger or weaker* connectivity in sensory and motor structures drives this failure requires additional statistical analyses, which is beyond the scope of this study. But these findings suggest that resistance to change is also likely caused by hypersensitivity to cues that promote SL, an effect that may well be tied to a hyperactive bias of the brain to conserve effort. Hence, a relevant question is whether this connectivity is associated with chronic health conditions such as arthritis of the hip/knee, muscle atrophy, and/or severe obesity—evidence of embodiment.

As suggested, our findings have important implications for the future of intervention research. First, consistent with the recent review by Verplanken and Orbell (2022), interventions targeting SL should be designed to *promote the acquisition of new habits*, or one's propensity to respond automatically to cues that led to the previous performance of a behavior. If ignored, there is a high probability that old habits will resurface upon completion of formal treatment. Second, understanding *resistance to changing sitting behavior and daily steps—*insights uncovered within the current study—are critical in evaluating readiness for change. Ideally, future research could develop methods that are less time intensive and resource dependent than neuroimaging and thus have greater clinical utility. Third, it is known that the formation of new habits is undermined when an individual is experiencing resource depletion, circumstances that need to be addressed as part of a comprehensive intervention (Verplanken and Orbell 2022). For older adults attempting to move away from SL, major sources of resource depletion can emerge from chronic health conditions, cognitive overload, depression, stress, and pain. Moreover, fourth, when possible, it is advisable to outsource behavioral control over habits to the environment (Maher and Conroy 2016). Hence, the inherent value of creating social and physical cues prompts movement across the day. The trick is that these cues need to be stable and consistent across time.

As noted at the beginning of this section, SL is a distinct class of behavior from EX; specifically, exercise typically involves a single bout of behavior, taking up a small portion of the day, whereas SL is the perfect inverse of one's *total daily activity profile*. Culturally and environmentally, we both reward and cue SL (Dempsey, Matthews, and Dashti 2020) and often punish older adults for engaging in EX by prescribing it in a manner that is aversive (Brand and Ekkekakis 2018). These characteristics of SL and exercise for older adults require creating new associations and habits for individualized *total daily activity profiles* that, while not precluding exercise, would benefit by adopting an approach rooted in self‐determination theory (Deci and Ryan 2008) (which guides motivational interviewing) that prioritizes diversity of movement and intrinsic motivation. In addition, a related issue is to underscore how important it is that in developing interventions with a focus on *total daily activity profiles*, conscious attention needs to be given to the impact of the proposed program on implicit associations with PA and PA‐related habits. For instance, if a new early morning, high‐intensity PA program fosters powerful negative associations with PA, then the person in question is likely to look forward to a relaxing afternoon on the couch, building *strong* habits for SL and creating potentially aversive feelings toward cues for increased PA (Brand and Ekkekakis 2018; Ekkekakis and Dafermos 2012).

Although this study provides novel insights into dynamic neural networks that are predictive of success with interventions that attempt to reduce sitting behavior and increase daily stepping, it has important limitations. First, the sample size was small, focused on older adults, and is restricted in socioeconomic and racial diversity, qualities that constrain the external validity of these results. Future studies on larger cohorts of older adults could address this concern. Second, the network components uncovered in the current study were extracted from baseline resting state scans. It would be instructive to examine potential changes in network structures from pre‐ to post‐intervention. This would be particularly valuable in examining the efficacy of interventions that target the neural deficits observed in the current study. Third, each network component is not simply a combination of regions that are more or less likely to be connected for the low or high groups. Each network component represents a more complex circuit that explains the connection variability in time and across participants. Thus, our approach does not allow for specifically determining the increased/decreased connectivity profiles as determinants of pre–post change in these outcomes. It is worth noting that our tensor‐based methodology applied to dynamic brain networks was blind to the outcome (time sitting or step counts) and only allowed identifying the networks with highest variability across brain connectivity, time, and participants. In other words, the components/networks were not extracted with respect to either time sitting or step counts. They were rather extracted in a data‐driven manner. The excellent prediction performance obtained via using these components/networks indicated their substantial contribution toward pre–post change in sitting time and step counts. But this does not mean the same role of each network and its underlying regions for both sitting time and step counts because SVM uses 289 features that characterize each component/network, and those features will have different loadings when predicting sitting time versus step counts. Future studies that use statistical analyses are needed to provide more insight about the specific roles of each region. We used the Shen atlas as it is one of the most widely used functional parcellations that would facilitate replicating our analyses on independent datasets to examine reproducibility. However, future studies need to examine the sensitivity of our results on the parcellation choice. Finally, in parcellating the brain into eight subnetworks in Figure 1, we assigned each node to a single subnetwork as previously described (Bahrami et al. 2022). We acknowledge that there is no perfect alignment of individual nodes to predefined subnetworks and that regions assigned to any specific network may be a part of other functional networks as well.

## 5 Conclusion

A sedentary lifestyle, marked by low daily activity volume and high levels of sedentary time, has enormous consequences on older adult's health. To our knowledge, this is the first study that identifies complex dynamic circuits in the brain that could underlie the variability in response to an intervention targeting daily activity and sitting behavior. Our results indicate that interactions between key regions of the DMN and the SMN play critical roles in mediating these behaviors among older adults, underscoring the importance of self‐regulatory capacity, as a well‐known determinant of one's daily activity and sedentary behaviors. This study demonstrates the promise of neural signatures in identifying the older adults who will succeed or fail to improve their active and sitting behaviors after an intervention, which could have important implications for designing individual‐centered interventions and targeted treatments.

## Author Contributions

**Mohsen Bahrami**: conceptualization, data curation, methodology, validation, visualization, writing–original draft, writing–review and editing, formal analysis, funding acquisition. **Jonathan H Burdette**: conceptualization, funding acquisition, writing–review and editing, investigation, data curation. **Paul J Laurienti**: conceptualization, investigation, writing—review and editing, methodology. **Barbara J Nicklas**: conceptualization, writing–review and editing, data curation. **W Jack Rejeski**: conceptualization, investigation, funding acquisition, writing—review and editing, validation, data curation. **Jason Fanning**: conceptualization, investigation, funding acquisition, writing–review and editing, methodology, project administration, data curation, supervision, resources, validation.

## Ethics Statement

The study protocol was approved by the Wake Forest School of Medicine Institutional Review Board.

## Consent

Documented informed consents and MRI compatibility information were obtained for all participants prior to scanning.

## Conflicts of Interest

The authors declare no conflicts of interest.

### Peer Review

The peer review history for this article is available at https://publons.com/publon/10.1002/brb3.70085

## Supporting information



Supporting Information

## Data Availability

The data used in this study may be provided upon reasonable request.
