# Supplementary material for: Neural Determinants of Sedentary Lifestyle in Older Adults: A Brain Network Analysis
Source: Brain Behav. 2025 Jan 8;15(1):e70085. doi: 10.1002/brb3.70085 (PMC11710895; doi:10.1002/brb3.70085)
Supplement: Supplementary file 1 — Supporting Information [file BRB3-15-e70085-s001.docx]

**Supplemental File**

**Tensor Decomposition Methodology.** The individual connectivity tensors across the $M_{Trn}$ ($M_{Trn}=28$) training samples were concatenated to construct a 4^th^ order connectivity tensor, $\mathcal{C}_{Trn}\in\mathbb{R}^{N\times N\times T\times M_{Trn}}$. Using tensor algebra, we can decompose $\mathcal{C}_{Trn}$ as follows

| $\mathcal{C}_{Trn}\mathcal{=G}\times_{1}\boldsymbol{U}^{\left( 1 \right)}\times_{2}\boldsymbol{U}^{\left( 2 \right)}\times_{3}\boldsymbol{U}^{\left( 3 \right)}\times_{4}\boldsymbol{U}^{\left( 4 \right)}$ | (S1) |
| --- | --- |

where $\times_{n}$ denotes the $n-mode$ tensor product, and $\boldsymbol{U}^{\left( n \right)}\in\mathbb{R}^{d_{n}\times d_{n}}$ is the singular vector matrix (or column space) in the $n^{th}$ mode with $d_{1}=d_{2}=N, d_{3}=T, and d_{4}=M_{Trn}$. Note that $\boldsymbol{U}^{\left( 1 \right)}$ and $\boldsymbol{U}^{\left( 2 \right)}$ will be equal in this study since connectivity matrices at each are symmetric. $\boldsymbol{U}^{\left( n \right)}$ can be further decomposed into $\boldsymbol{U}^{\left( n \right)}=[\boldsymbol{u}_{1}^{\left( n \right)} \boldsymbol{u}_{2}^{\left( n \right)} \boldsymbol{u}_{3}^{\left( n \right)} \ldots\boldsymbol{u}_{d_{n}}^{(n)}]$ where $\boldsymbol{u}_{i}^{\left( n \right)}\in\mathbb{R}^{d_{n}\times1}, i\in\left\{ 1, 2, 3,\ldots, d_{n} \right\}$ is the $i^{th}$ singular vector in the $n^{th}$ mode. $\mathcal{G\in}\mathbb{R}^{N\times N\times T\times M_{Trn}}$ is the core tensor, which can be further decomposed into orthogonal sub-tensors, $\mathcal{G}_{k}^{(n)}$ with Frobenius norms, $\sigma_{k}^{(n)}=\left| \mathcal{G}_{k}^{(n)} \right|$ by fixing $n$ at each $k\in\{1, 2, 3, \ldots, d_{n}\}$.

Elements of $\mathcal{C}_{Trn}$, represented by $c_{ijtfl}$, can be obtained as follows

| $c_{ijtfl}=\sum_{pqrm} u_{ip}^{(1)}u_{jq}^{(2)}u_{tr}^{(3)}u_{lm}^{(4)}g_{pqrm}=\sum_{pqrm} {(u}_{ip}^{(1)}u_{jq}^{(2)}){g_{pqrm}(u}_{tr}^{(3)}u_{lm}^{(4)})$ | (S2) |
| --- | --- |

where $u_{uv}^{(n)}$ denotes the $(u,v)$ element of $\boldsymbol{U}^{(n)}$, and $g_{pqrm}$ denotes the $(p,q,r,m)$ element of $\mathcal{G.}$Eq. S1 can be expanded as follows

| $\mathcal{C}_{Trn}=\sum_{i=1}^{N} \sum_{j=1}^{N} \sum_{t=1}^{T} \sum_{l=1}^{M_{Trn}} \boldsymbol{(u}_{i}^{\left( 1 \right)} \bigotimes\boldsymbol{u}_{j}^{\left( 2 \right)})g_{ijtl}(\boldsymbol{u}_{t}^{\left( 3 \right)} \boldsymbol{\bigotimes u}_{l}^{\left( 4 \right)})$ | (S3) |
| --- | --- |

where $\bigotimes$denotes the Kronecker product, and $\boldsymbol{u}_{i}^{\left( n \right)}$ denotes the $n^{th}$ mode $i^{th}$ singular vector of $\boldsymbol{U}^{\left( n \right)}$ as described above. Eq. S3 can be rewritten in matrix form as follows

| $\boldsymbol{C}_{Trn}=\left( \boldsymbol{U}^{\left( 1 \right)}\boldsymbol{\bigotimes}\boldsymbol{U}^{\left( 2 \right)} \right)\boldsymbol{G}{\boldsymbol{(}\boldsymbol{U}^{\left( 3 \right)}\boldsymbol{\bigotimes}\boldsymbol{U}^{\left( 4 \right)}\boldsymbol{)}}^{T}$ | (S4) |
| --- | --- |

where $\boldsymbol{C}_{Trn}\in\mathbb{R}^{N^{2}\times{TM}_{Trn}}$ and $\boldsymbol{G}\in\mathbb{R}^{N^{2}\times{TM}_{Trn}}$ denote the matrix forms of $\mathcal{C}_{Trn}$ and $\mathcal{G}$, respectively. Similar to a singular value decomposition (SVD), in which the single values are in descending order, and represent the amount of variance captured by the corresponding singular vectors, Frobenius norms, $\sigma_{k}^{(n)}$, of subtensors, $\mathcal{G}_{k}^{(n)}$, are in descending order as well, and show the amount of variance captured by their corresponding $n^{th}$ singular vector matrices. Thus, as in the case with SVD, $\mathcal{C}_{Trn}$ can be approximated by the singular vector matrices and core tensors which maintain the highest variability in $\mathcal{C}_{Trn}$. This will allow for performing a *truncated* decomposition of $\mathcal{C}_{Trn}$, with substantially lower ranks. As shown in the literature, the optimum solution for a truncated decomposition is obtained when the core tensor, $\mathcal{G}$, is in a hypercube form, $\mathcal{G\in}\mathbb{R}^{R\times R\times R\times R}$, where $R\leq\min\left\{ N, N, T, M_{Trn} \right\}$ [46]. In our study, $R$ was set to 28 given that $\min\left\{ N, N, T, M_{Trn} \right\}=M_{Trn}=28$. Using the truncated tensor, eqs. S3, S4 can be rewritten as follows:

| $\mathcal{C}_{Trn}=\sum_{i=1}^{R} \sum_{j=1}^{R} \sum_{t=1}^{R} \sum_{l=1}^{R} \boldsymbol{(u}_{i}^{\left( 1 \right)} \bigotimes\boldsymbol{u}_{j}^{\left( 2 \right)})g_{ijtl}(\boldsymbol{u}_{t}^{\left( 3 \right)} \boldsymbol{\bigotimes u}_{l}^{\left( 4 \right)})$ | (S5) |
| --- | --- |

| $\boldsymbol{C}_{Trn}=\left( \boldsymbol{U}_{r}^{\left( 1 \right)}\boldsymbol{\bigotimes}\boldsymbol{U}_{r}^{\boldsymbol{(2)}} \right) \boldsymbol{G}_{r} {\boldsymbol{(}\boldsymbol{U}_{r}^{\boldsymbol{(}3\boldsymbol{)}}\boldsymbol{\bigotimes}\boldsymbol{U}_{r}^{\boldsymbol{(4)}}\boldsymbol{)}}^{T}$ | (S6) |
| --- | --- |

Where $\mathcal{C}_{Trn}\in\mathbb{R}^{R\times R\times R\times R}$ is the lower rank connectivity tensor, and $\boldsymbol{C}_{Trn}\in\mathbb{R}^{R^{2}\times R^{2}}$ is the lower rank connectivity tensor in matrix form, in which $\boldsymbol{U}_{r}^{\left( 1 \right)}\boldsymbol{,}\boldsymbol{U}_{r}^{\left( 2 \right)}\boldsymbol{\in}\mathbb{R}^{N\times R}$, $\boldsymbol{U}_{r}^{\left( 3 \right)}\boldsymbol{\in}\mathbb{R}^{T\times R}$, $\boldsymbol{U}_{r}^{\left( 4 \right)}\boldsymbol{\in}\mathbb{R}^{M_{Trn}\times R}$, and $\boldsymbol{G}_{r}\boldsymbol{\in}\mathbb{R}^{R\times R}$. The Kronecker product of the first two modes of $\boldsymbol{C}_{Trn}$, i.e., $\boldsymbol{U}_{r}^{\left( 1 \right)}\boldsymbol{\bigotimes}\boldsymbol{U}_{r}^{\boldsymbol{(2)}}$**,** represents the connectivity space, and thus the projection of the training samples across time onto this reduced rank connectivity space, can be obtained as follows

| $\boldsymbol{C}_{rTrn}=\boldsymbol{G}_{r} {\boldsymbol{(}\boldsymbol{U}_{r}^{\boldsymbol{(}3\boldsymbol{)}}\boldsymbol{\bigotimes}\boldsymbol{U}_{r}^{\boldsymbol{(4)}}\boldsymbol{)}}^{T}$ | (S7) |
| --- | --- |

where $\boldsymbol{C}_{rTrn}\in\mathbb{R}^{R^{2}\times TM_{Trn}}$ is the matrix form of the reduced-rank connectivity tensor of the training samples across time and frequency. We can then use the reduced rank connectivity space, $\boldsymbol{U}_{r}^{\left( 1 \right)}\boldsymbol{\bigotimes}\boldsymbol{U}_{r}^{\boldsymbol{(2)}}$, to reduce the rank of the test sample’s connectivity tensor, $\boldsymbol{C}_{Tst}\in\mathbb{R}^{N^{2}\times TM_{Tst}}$, as follows

| $\boldsymbol{C}_{rTst}={\boldsymbol{(}\boldsymbol{U}_{r}^{\left( 1 \right)}\boldsymbol{\bigotimes}\boldsymbol{U}_{r}^{\boldsymbol{(}2\boldsymbol{)}}\boldsymbol{)}}^{\boldsymbol{-}1} \boldsymbol{C}_{Tst}$ | (S8) |
| --- | --- |

Where $\boldsymbol{C}_{rTst}\in\mathbb{R}^{R^{2}\times TM_{Tst}}$ is the matrix form of the reduced-rank connectivity tensor of the test samples across time. Finally, we can reshape $\boldsymbol{C}_{rTrn}$ and $\boldsymbol{C}_{rTst}$ into tensor forms, $\mathcal{C}_{rTrn}\in\mathbb{R}^{R\times R\times T\times M_{Trn}}$ and $\mathcal{C}_{rTst}\in\mathbb{R}^{R\times R\times T\times M_{Tst}}$, and average them across time and frequency to obtain the summarized reduced-rank $R\times R$ connectivity tensor for each participant. Such reduced-rank connectivity tensors with embedded time information, explain the variability across participants. We thus used the final $R\times R$ connectivity tensors for our prediction analysis.

The principal vectors of either one of the first two modes (due to symmetric correlation matrices) can be used to reconstruct the brain network components associated with those principal vectors. For instance, using $\boldsymbol{u}_{i}^{\left( 1 \right)}$ and $\boldsymbol{u}_{j}^{\left( 1 \right)}$, the network component associated with these vectors, $\boldsymbol{S}_{ij}\in\mathbb{R}^{N\times N},$ can be reconstructed through outer product of these two vectors, i.e., $\boldsymbol{S}_{ij}\boldsymbol{= u}_{i}^{\left( 1 \right)}\odot\boldsymbol{u}_{j}^{\left( 1 \right)}$. However, as in the case with SVD, the diagonal elements of the core tensor carry the highest variance, and thus the network components associated with $i=j$ will yield network components which comprise the highest information. As such and for simplicity, we only present network components associated with the diagonal elements of the core tensor.

**HOSVD Decomposition.** Using a rank of 28 ($R=28$ in eq. S5), our HOSVD analysis decomposed each mode into a lower-rank data with 28 components, including: $\boldsymbol{U}_{r}^{\left( 1 \right)}\boldsymbol{,}\boldsymbol{U}_{r}^{\left( 2 \right)}\boldsymbol{\in}\mathbb{R}^{268\times28}$ for connectivity, $\boldsymbol{U}_{r}^{\left( 3 \right)}\boldsymbol{\in}\mathbb{R}^{T\times28}$ for time ($T=137$for the window lengths of $61$), and $\boldsymbol{U}_{r}^{\left( 5 \right)}\boldsymbol{\in}\mathbb{R}^{28\times28}$ for subjects. Fig S1 shows the variance explained by each component (i.e., singular vector) for connectivity, time, and subjects for the first 20 components. As mentioned earlier, the variance was determined using the square of the singular values (i.e., Frobenius norms) associated with each component. We then normalized each component’s variance to the variance of the first component and averaged across the 100 permutations. It is important to note that although we had different groupings for the $st$ and $ns$ which entailed using different training samples for each permutation in our tensor decomposition analyses, the final components were almost the same for the two as we averaged across the 100 permutations. In other words, our tensor decomposition analysis was almost data-driven and independent of the grouping labels. The difference between the two was embedded in the SVM weights during our prediction analysis learning.

**
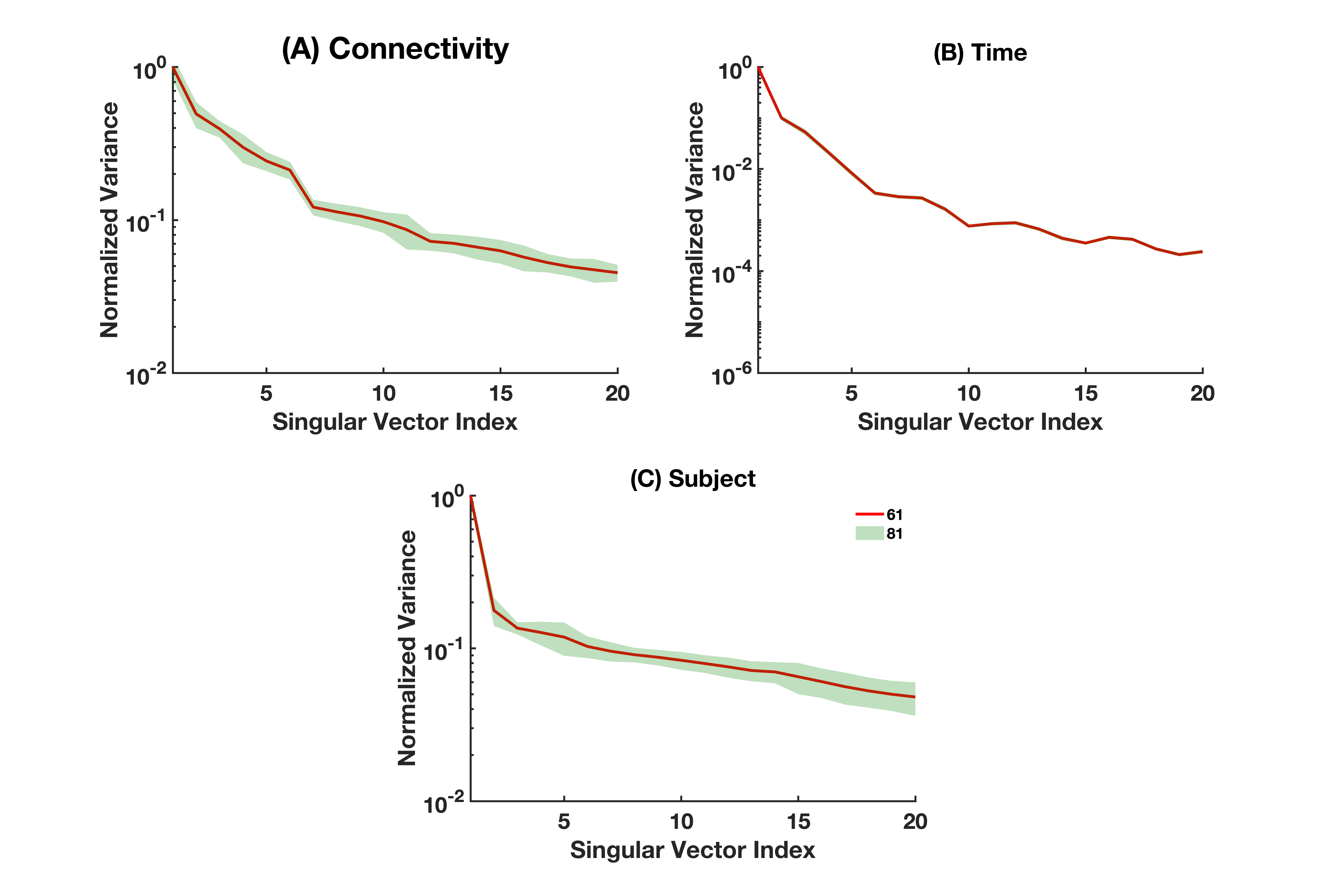
**

**Figure S1. Component variance across different modes.** Normalized variance explained by each component across the four included modes including: Connectivity, Time, and Individual Subjects are shown in **A, B**, and **C**, respectively, for the networks thresholded at 10% density and the window size of 61 $TRs$. The variance shows the average Frobenius norms across the 100 permutations. Variance norms are normalized to the variance of the first component. We have also shown the standard deviation ($\pm3\times sd$) of the normalized variances (shaded areas)*.* This figure shows the average variance across the 100 permutations for each mode.

**Performance Measurements.** Note that despite training the linear SVM with binary labels, the predicted labels were not binary and were instead two probability values of belonging to each group. For each sample, we used the group with the higher probability as the predicted group for that sample. However, since higher probability does not always imply a higher confidence in group assignment, we computed the cross entropy of the prediction model as well to ensure that predicted probabilities were close to the true binary labels. We used the same method as in [28, 48], and have provided more detail below. Let $a_{id}$denote the binary label for sample (i.e., participant in our study) $i$ where $d\in\{1 , \ldots,D\}$ represents the group with $D=2$ in our study. Thus, $a_{id}=1$ if sample $i$ is in group $d$ and zero otherwise. If $a_{id}^{'}$ denotes the predicted probability of sample $i$ belonging to group $d$, then we can use the following equation to obtain the average cross-entropy ($CE$) across the entire test samples, $M_{Tst}$ for the permutation fold $p$

$${CE}_{p}=-\frac{1}{M_{Tst}}\sum_{i=1}^{M_{Tst}} \sum_{d}^{D} \left( a_{id}\ln\left( a_{id}^{'} \right) + \left( 1-a_{id} \right)\ln\left( {{1-a}}_{id}^{'} \right) \right) (S9)$$

Also, for each permutation $p\in1, 2, 3, \ldots, 100$, using the equations below, we determined the accuracy (${Acc}_{p}$), sensitivity (${Sens}_{p}$), and specificity (${Spec}_{p}$), on test samples.

$$\left\{ \begin{matrix} {Acc}_{p}=\frac{Total number of correctly predicted test samples}{Total number of samples} \\ {Sens}_{p}=\frac{Number of correctly predicted samples from low-st (/ns)}{Total number of samples from low-st (/ns)} \\ {Spec}_{p}=\frac{Number of correctly predicted samples from high-st (/ns)}{Total number of samples from high-st (/ns)} \end{matrix} \right. (S10)$$

Finally, we averaged across the 100 permutations to obtain the final performance measures,

$Acc=\frac{{Acc}_{1}+\ldots+ {Acc}_{100}}{100}$ , $Sens=\frac{{Acc}_{1}+\ldots+ {Acc}_{100}}{100}$ , $Spec=\frac{{Acc}_{1}+\ldots+ {Acc}_{100}}{100}$, and $CE=\frac{{CE}_{1}+\ldots+ {CE}_{100}}{100}$

The bar plots in Figure 2 show Acc, Sense, and Spec. In Figure S2 below, we have added the CE as well. Note that lower CE shows higher confidence in prediction.


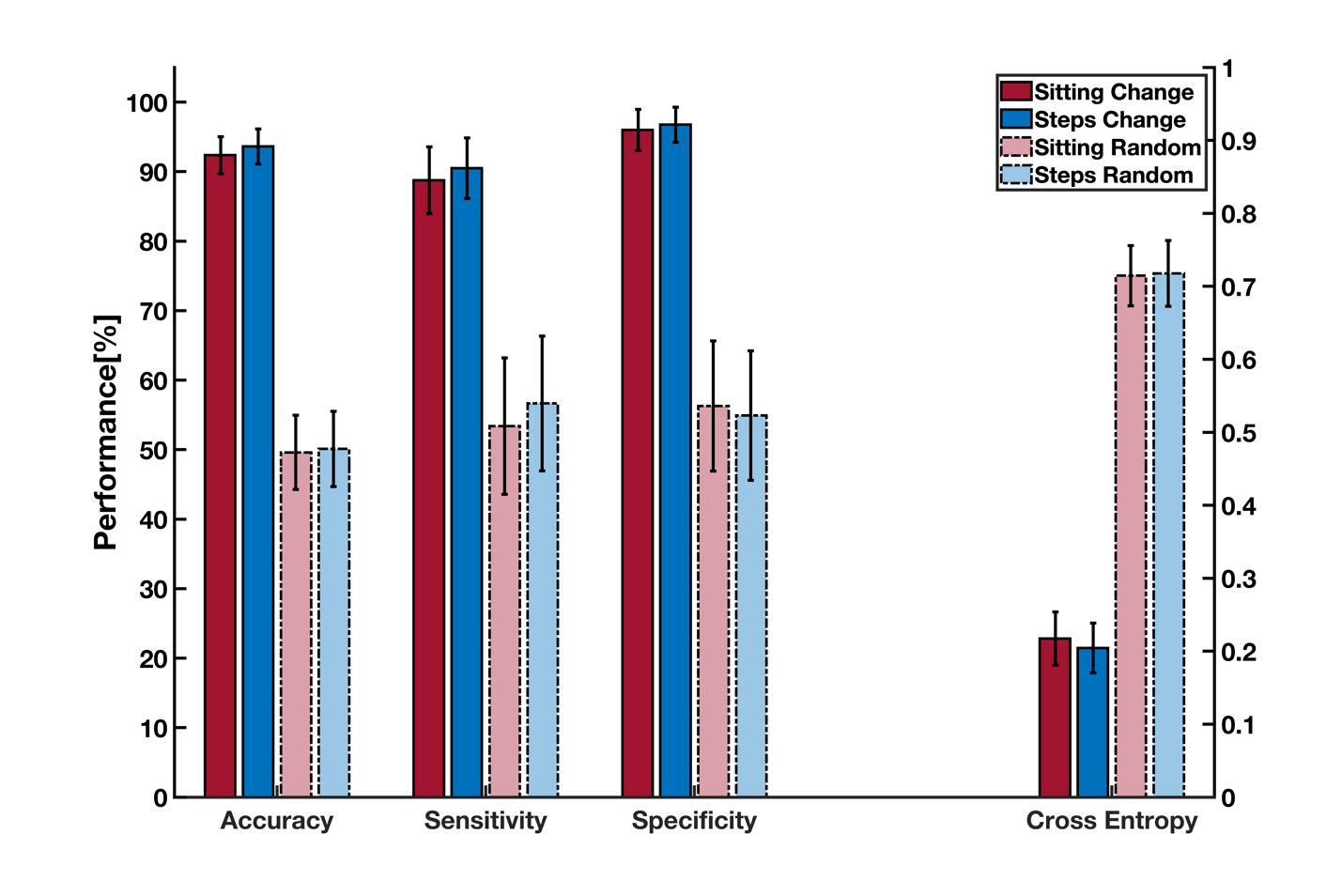


**Figure S2. Average prediction performance across 100 permutations.** Average accuracy, sensitivity, specificity, as well as the average cross-entropy are shown for the main analyses and the analyses with random group assignments. As in Figure 2, the average and the standard deviation across the 100 permutations are shown in this figure.

**Table S1.** Key Demographics of the parent study and the subsample used in our study

| Variable | Parent Study (N = 63) | Current Study (N = 36) | p-value |
| --- | --- | --- | --- |
| Age | $\boldsymbol{70.38\pm5.48}$ | $\boldsymbol{70.42\pm5.06}$ | **0.96** |
| Baseline Weight (kg | $\boldsymbol{95.14\pm11.58}$ | $\boldsymbol{93.96\pm11.14}$ | **0.62** |
| Male/Female | $\boldsymbol{45/18}$ | $\boldsymbol{29/7}$ | **0.31** |

As table S1 shows, the sample used in our study represents the parent scanning cohort as their key characteristics don’t have any significant differences with those of the parent study population. Unfortunately, data for 8 participants from the parent study were not available (we used 63 rather than 71) but we are confident that including those participants would not change the stats presented below significantly. We used t-test to compare Age and Baseline weight and chi-squared test to compare the Male/Female Ratio.

**Statistical Power Analysis.** Since our study uses machine learning rather than a standard statistical methodology to predict pre-post changes in SB and then maps the extracted features on brain space, the power should be defined with respective to the confidence in the prediction accuracy. We used 100 permutations of random cross-validation to ensure that our results are reliable and further used that same permutation but with shuffling the labels across the two groups to compare our results to random classification. As Figure 2 shows, when using predicting the pre-post change in low- versus high-change, our prediction accuracy exceeds 92%. However, when using random labeling, the accuracy is around 50%.

Table S2 shows the effect sizes for 5% or 10% increase in the average accuracy across 100 permutations and varying standard deviations (SD). Table S3 shows the achieved statistical power for small, medium, and large effect sizes according to the Cohen’s d definitions(Cohen, 1988). The threshold to achieve 80% power is 0.28.

| **Table 2.** Statistical Power   \| Effect Size \| \| Effect Magnitude \| \| Power \| \| --- \| --- \| --- \| --- \| --- \| \| 0.2 \| Small \| \| 0.5 \| \| \| 0.5 \| Medium \| \| 0.99 \| \| \| 0.8 \| large \| \| 1 \| \| | **Table 3** Effect Sizes   \| Mean 1 \| Mean 2 \| SD \| Cohen’s d \| \| --- \| --- \| --- \| --- \| \| 80 \| 85 \| 20 \| 0.25 \| \| 80 \| 85 \| 10 \| 0.5 \| \| 80 \| 90 \| 15 \| 0.67 \| |
| --- | --- | --- | --- | --- | --- | --- | --- | --- | --- | --- | --- | --- | --- | --- | --- | --- | --- | --- | --- | --- | --- | --- | --- | --- | --- | --- | --- | --- | --- | --- | --- | --- | --- | --- | --- | --- | --- |

A difference of over 40% with the classification that used random labeling shows great statistical power for the obtained accuracies.
